# Spin-momentum coupled Bose-Einstein condensates with lattice band pseudospins

**DOI:** 10.1038/ncomms10867

**Published:** 2016-02-29

**Authors:** M. A. Khamehchi, Chunlei Qu, M. E. Mossman, Chuanwei Zhang, P. Engels

**Affiliations:** 1 Department of Physics and Astronomy, Washington State University, Pullman, Washington 99164, USA; 2 Department of Physics, The University of Texas at Dallas, Richardson, Texas 75080, USA

## Abstract

The quantum emulation of spin-momentum coupling, a crucial ingredient for the emergence of topological phases, is currently drawing considerable interest. In previous quantum gas experiments, typically two atomic hyperfine states were chosen as pseudospins. Here, we report the observation of a spin-momentum coupling achieved by loading a Bose-Einstein condensate into periodically driven optical lattices. The *s* and *p* bands of a static lattice, which act as pseudospins, are coupled through an additional moving lattice that induces a momentum-dependent coupling between the two pseudospins, resulting in *s*–*p* hybrid Floquet-Bloch bands. We investigate the band structures by measuring the quasimomentum of the Bose-Einstein condensate for different velocities and strengths of the moving lattice, and compare our measurements to theoretical predictions. The realization of spin-momentum coupling with lattice bands as pseudospins paves the way for engineering novel quantum matter using hybrid orbital bands.

## Abstract (web summary)

The optical trapping of ultracold atoms allows for the simulation and controlled exploration of phenomena normally found in condensed matter systems. Here, the authors demonstrate spin–orbit coupling between lattice band pseudospins in a Bose-Einstein condensate of ultracold atoms.

## Graphical abstract

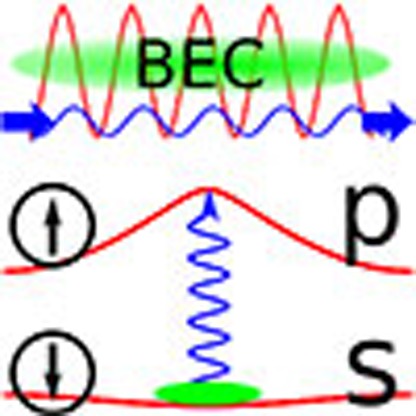

Spin-momentum coupling (SMC), commonly called spin–orbit coupling, is a crucial ingredient for many important condensed matter phenomena such as topological insulator physics, topological superconductivity, spin Hall effects and so on^1^,^2^,^3^. In this context, the recent experimental realization of SMC in ultracold atomic gases provides a powerful platform for engineering many interesting and novel quantum phases^4^,^5^,^6^,^7^,^8^,^9^. In typical experiments, two atomic hyperfine states act as two pseudospins that are coupled to the momentum of the atoms through stimulated Raman transitions^10^,^11^. However, ultracold atoms in optical lattice potentials possess other types of degrees of freedom, which can also be used to define pseudospins^12^,^13^. A natural and important question is whether such new types of pseudospins can be employed to generate SMC.

In optical lattices filled with ultracold atoms, *s*- and *p*-orbital bands are separated by a large energy gap and can be defined as two pseudospin states. One significant difference between hyperfine state pseudospins and lattice band pseudospins lies in the energy dispersion of spin-up and spin-down orientations: the dispersion relations are the same for hyperfine state pseudospins, while they are inverted for lattice band pseudospins. It is well known from topological insulators and superconductor physics that inverted band dispersions, together with SMC, play a central role for topological properties of materials^14^,^15^,^16^. Therefore, it is natural to expect that the inverted band pseudospins, when coupled with the lattice momentum, may lead to interesting topological phenomena in cold atomic optical lattices. Recent experiments with shaken optical lattices (that is, lattices in which the lattice sites are periodically in time shifted back and forth^17^) have realized a simple coupling (Ω*σ*_*x*_ coupling, where Ω is the coupling strength and *σ*_*x*_ a Pauli matrix) between *s*- and *p*-band pseudospins, analogous to Rabi coupling between two regular spins^18^. However, for the exploration of exotic phenomena in optical lattice systems, such as Fulde–Ferrell–Larkin–Ovchinnikov (FFLO) phases^19^,^20^ and Majorana fermions^16^, SMC with *s*- and *p*-band pseudospins is highly desirable^21^,^22^,^23^,^24^.

In our experiments we realize such *s*–*p* band SMC for a Bose-Einstein condensate (BEC) using a weak moving lattice to generate Raman coupling between *s*- and *p*-band pseudospins of a static lattice^25^. The moving lattice acts as a periodic driving field^26^,^27^,^28^,^29^,^30^,^31^ and has previously been used to generate an effective magnetic field in the lowest *s* band of a tilted optical lattice^32^,^33^. In our experiment, the driving frequency of the moving lattice is chosen close to the energy gap between *s* and *p* bands at zero quasimomentum, leading to a series of hybrid *s*-*p* Floquet-Bloch (FB) band structures. FB band structures in optical lattices give rise to interesting and important phenomena in cold atoms and solids^34^,^35^, as is evidenced by the recent experimental realization of a topological Haldane model in a shaken honeycomb optical lattice^36^, and the observation of FB states on the surface of a topological insulator^37^. We show that the moving lattice generates two types of coupling between *s-* and *p*-band pseudospins: a momentum-independent Rabi coupling (Ω*σ*_*x*_) and SMC (*ασ*_*x*_ sin(*q*_*x*_*d*), where *q*_*x*_ is the quasimomentum and *d* the lattice period), with strengths of the same order. The coexistence of these two types of coupling leads to asymmetric FB band dispersions^38^. We investigate the FB band structures by measuring the quasimomentum of the BEC. The initial phase of the moving lattice plays a significant role in the Floquet dynamics^29^, the effects of which are explored through a quantum quench-induced dynamical coupling of the FB bands. Results are compared with the theoretical predictions from a simple two-band model and from numerical simulations of the Gross–Pitaevskii (GP) equation.

## Results

### Experimental set up

**Figure 1:**
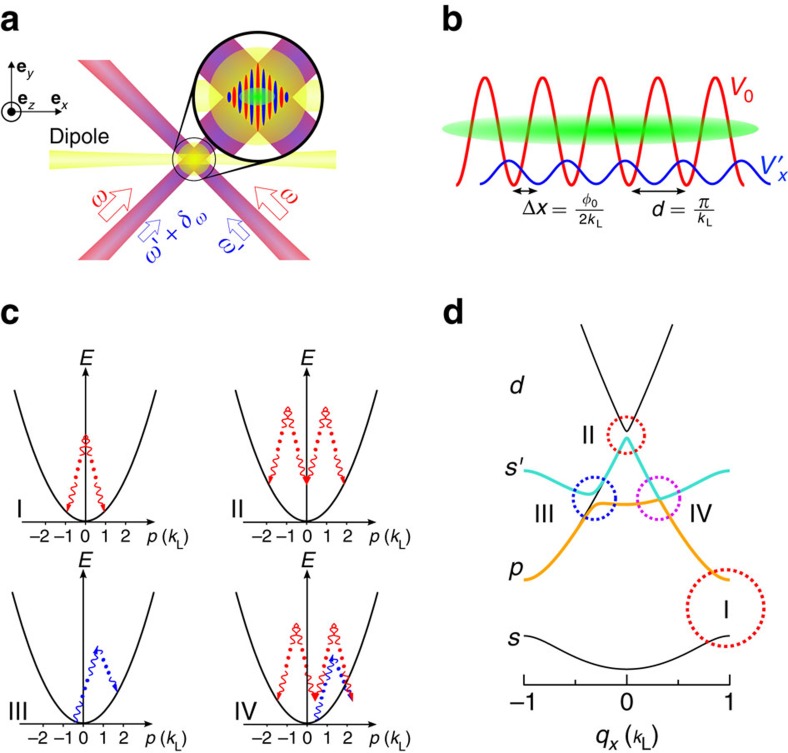
Experimental set up and schematic lattice illustration. (**a**) Experimental arrangement. The crossed dipole trap beams propagate in the **e**_*x*_ and **e**_*z*_ directions. The static and moving lattices have overlapping beams propagating along **e**_*x*_+**e**_*y*_ and −**e**_*x*_+**e**_*y*_. The static lattice is generated using the red beams with laser frequency *ω*, and moving lattice is generated using the blue beams with laser frequencies *ω*′ and *ω*′+*δω*. (**b**) Lattice potentials along the **e**_*x*_ direction. The lattice period *d* is identical for the static lattice *V*_0_ and the moving lattice 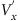. The initial offset between lattice sites of the static and moving lattice, Δ*x*, is dependent on the initial phase *φ*_0_ between the two lattices. (**c**,**d**) Illustration of the multi-photon processes for the driven lattice system and the corresponding FB band structure in the first Brillouin zone. The static lattice induces a large energy gap (I) through a two-photon process and a small energy gap (II) through a four-photon process. The moving lattice induces an energy gap when the *s* band and the *p* band are coupled through (III). A smaller energy gap is produced by a combination of the static and moving lattice (IV).

To generate the *s*–*p* band SMC and FB band structures, we begin with a ^87^Rb BEC composed of ∼5 × 10^4^ atoms confined in a crossed dipole trap. A static lattice is generated by two perpendicular laser beams with wavelength *λ*≈810 nm intersecting at the position of the BEC, as schematically shown in Fig. 1a. The harmonic trap frequencies due to the envelope of the static lattice beams and the crossed dipole trap are (*ω*_*x*_, *ω*_*y*_, *ω*_*z*_)=2*π* × (41, 159, 115) Hz, where **e**_*x*_ points along the lattice, **e**_*y*_ is the horizontal transverse direction and **e**_*z*_ is the vertical direction. A weak moving lattice with the same lattice period as the static lattice, *d*=*π*/*k*_L_ where 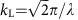, is then overlaid with the static lattice (Fig. 1b). The moving lattice beams are ∼180 MHz detuned from the static lattice. A small frequency difference Δ_*ω*_ between the two moving lattice beams determines the velocity of the lattice according to *v*_lattice_=Δ_*ω*_/2*k*_L_. To induce *s*–*p* orbital band coupling, |Δ_*ω*_| is chosen close to the energy gap *E*_*sp*_ between the *s* and *p* bands of the static lattice at quasimomentum *q*_*x*_=0.

One outstanding feature of the coupling scheme employed in these experiments is the asymmetry of the effective *s*–*p* FB bands, which exhibit a local minimum located at a finite quasimomentum *q*_*x*_≠0. The direction in which the minimum is shifted away from *q*_*x*_=0 is determined by the sign of Δ_*ω*_ (which determines the direction of motion of the moving lattice) and |Δ_*ω*_|−*E*_*sp*_ (that is, the detuning of the drive from the bandgap at *q*_*x*_=0). Before describing experimental results and a formal derivation of the band structure using Floquet theory^29^,^30^, we lay the groundwork by presenting a multi-photon resonance picture that provides intuitive insights (Fig. 1c,d). In this picture, one starts with the parabolic dispersion of a free atom in the absence of any external potentials. An optical lattice then induces 2*n*-photon couplings (with *n* being an integer number) between points of the dispersion relation due to the absorption and stimulated emission processes. The couplings are centred around pairs of points that fulfil conservation of energy and momentum. At these points, bandgaps open due to avoided crossings. Examples for possible couplings due to the static lattice (red arrows in Fig. 1c) and the moving lattice (blue arrows in Fig. 1c), and the associated bandgaps in the first Brillouin zone are shown in Fig. 1. Different coupling strengths lead to different sizes of bandgaps, which result in an asymmetric band structure.

In another pictorial way, the Floquet band structure for the time-periodic system can be constructed by creating multiple copies of the Bloch band structure of the static lattice that are offset in energy by |Δ_*ω*_|. The moving lattice couples the *p* band and the shifted *s* band (labelled by *s*′ in Fig. 1d) at points where the shifted *s* band intersects the unshifted *p* band. The gaps opened by the coupling can formally be calculated using Floquet theory.

### Experimental measurements

Adiabatic loading of the BEC into an *s*–*p* FB band is achieved by first ramping on the intensity of the static lattice, followed by adiabatically ramping on the moving lattice intensity. In this procedure, the initial relative phase between the two lattices, *φ*_0_ (Fig. 1b), becomes irrelevant and can effectively be set to zero. As we shall show in this paper, if the moving lattice is suddenly jumped on instead of adiabatically ramped on, this initial relative phase may manifest itself by markedly changing the dynamics of the system^29^.

**Figure 2:**
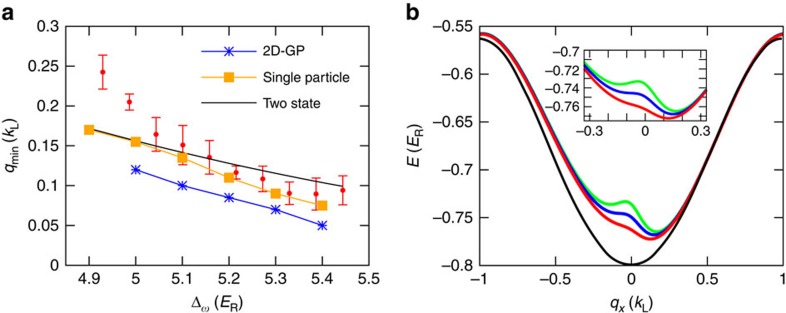
Effects of the driving frequency. (**a**) Band minimum *q*_min_ for the upper hybrid band versus driving frequency Δ_*ω*_. The depth of the moving lattice is 1 *E*_R_. The filled circles are experimental measurements averaged over 5 data points with s.d. errorbars. The black line shows the theoretical prediction of a two-band model. The squares and stars are the results of numerical simulations of the Schrödinger equation and the GP equation, respectively. (**b**) Calculated upper hybrid *s*–*p* FB band structure for different driving frequencies Δ_*ω*_=4.99 *E*_R_, 5.1 *E*_R_ and 5.22 *E*_R_ from top to bottom. The lowest (black) curve is the *s*-orbital band without the presence of the driving field.

Figure 2a shows the measurement of the band minimum, *q*_min_, for different driving frequencies, Δ_*ω*_, after adiabatically loading a BEC into a FB band. The driving frequencies are chosen such that *ħ*Δ_*ω*_ lies in the gap at *q*_*x*_=0 between the *p* band (4.64 *E*_R_, where 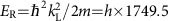 Hz) and the *d* band (5.44 *E*_R_). After adiabatically loading a BEC into a FB band, all lasers are switched off and the BEC is imaged after 14 ms time-of-flight (TOF). In the experimental images clouds with three different kinetic momenta are seen: *q*_min_ and *q*_min_±2*k*_L_. The kinetic momentum of the middle component, *q*_min_, is equal to the quasimomentum of the BEC in the hybrid *s*–*p* band. Therefore the quasimomentum can be obtained by measuring the position of the three components in the TOF images^39^. Each data point in Fig. 2a is an average over five iterations of the measurement. A shift of the quasimomentum is detected that decreases with increasing driving frequency (Fig. 2a) as the coupling between the *p* band and shifted *s* band becomes weaker. The observed shift indicates a shift of the minimum of the upper hybrid band (Fig. 2b) into which the BEC is adiabatically loaded. The solid line in Fig. 2a shows *q*_min_ calculated from a simple two-band model (see below) and is in reasonable agreement with the data. The symbols are the results from solution of the Schrödinger equation (squares) and the GP equation (stars), with finite nonlinear interaction strength. The periodically driven dynamics are simulated using the time-dependent GP equation for a two-dimensional system with the same geometry as the experiment. The width of the BEC is ∼10 μm. We see that the interaction could modify the single-particle results. Since the atom loss is usually large in the experiment, the numerical results are not intended for direct comparison with the measurements. See Methods for some samples of the GP simulations.

**Figure 3:**
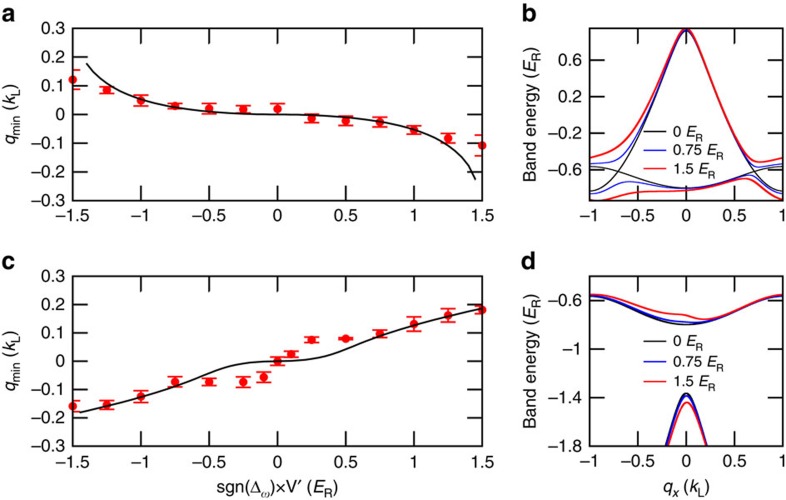
Effects of the driving strength. Band minimum *q*_min_ versus driving field strength *V*′ for different driving frequencies of (**a**) |Δ_*ω*_|=2.92 *E*_R_ and (**c**) |Δ_*ω*_|=5.21 *E*_R_. The red points are experimental data averaged over 5 data points with s.d. errorbars, and the solid lines are the the theoretical predictions from a two-band model. *sgn*(Δ_*ω*_) determines the direction of motion of the moving lattice. (**b**,**d**) Corresponding hybrid band structures for different driving field strengths 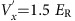, 0.75 *E*_R_ and 0 *E*_R_ (red, blue and black curves, respectively).

Figure 3 presents a complementary data set for which the driving frequency is set to a constant value with |Δ_*ω*_|<*E*_*sp*_ (Fig. 3a) or |Δ_*ω*_|>*E*_*sp*_ (Fig. 3c) and the quasimomentum is determined for various depths of the moving lattice. The sign of Δ_*ω*_ determines the direction of motion of the moving lattice. For |Δ_*ω*_|<*E*_*sp*_ the BEC resides in the lower hybrid *s*–*p* FB band (Fig. 3b), while for |Δ_*ω*_|>*E*_*sp*_ it is in the upper hybrid band (Fig. 3d). This leads to a shift of the *q*_min_ into opposite directions for the two cases. For a given driving frequency, the coupling of the two bands is stronger for larger driving field strength (that is, larger depth of the moving lattice) so that the BEC is shifted to a larger absolute value of quasimomentum.

**Figure 4:**
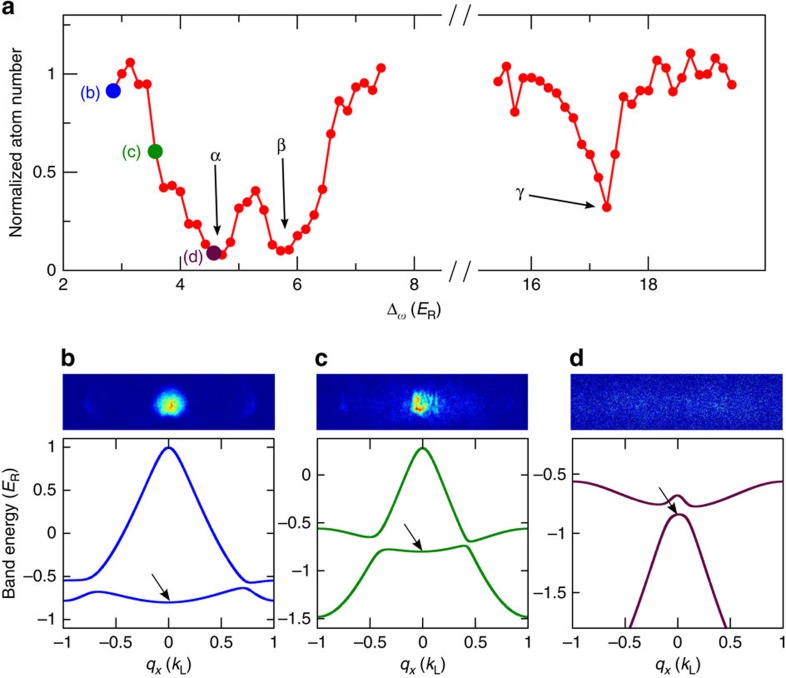
Stability of the Floquet system. (**a**) Number of atoms remaining in trap after adiabatically loading a BEC into the FB band, normalized to initial atom number determined from independent experimental runs. The static lattice is ramped on to 5.47 *E*_R_ in 200 ms. Then the moving lattice is ramped on to a depth of *V*′=0.5 *E*_R_ in 60 ms. The dips *α*, *β* and *γ* occur close to the Bloch bands *p*, *d* and *f*. (**b**–**d**) The TOF images and the effective band structures for data points labelled in **a**. The arrows show where the BEC is situated after the moving lattice is adiabatically ramped on.

Floquet systems such as the one in our experiment are described by quasienergy bands. They do not have a thermodynamic ground state, and in the presence of many-body interactions their stability can be affected by a variety of factors^40^,^41^,^42^. Resonance–induced collective excitations and modulational instabilities can lead to losses^43^. A prominent feature of band inversion is the fact that hybrid bands can transition between stable and modulationally unstable structures. As shown in Fig. 4a, when the driving frequency approaches the dip *α* from the left, the lower hybrid band evolves from a globally stable structure to a locally stable structure and eventually enters the globally unstable region at the quasimomentum where BEC mainly resides (Fig. 4b). The instability causes excitations and heating of the BEC. Experimentally, we study the stability of the system by determining the number of condensed atoms left in the trap after the static and the moving lattices are successively and adiabatically ramped on. As the instabilities cause heating, the heated atoms are evaporated out of the trap. Therefore absorption imaging reveals an atom loss from the BEC as shown in Fig. 4. The dips *α*, *β* and *γ* in Fig. 4a occur when the driving frequency is chosen such that it leads to a coupling close to the Bloch bands *p*, *d* and *f* of the static lattice at *q*_*x*_=0, respectively.

### Minimal two-band model

The dynamics of the BEC are governed by the full time-dependent GP equation, 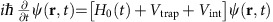 where *V*_trap_ and *V*_int_ are the external trapping potential and the mean-field interaction, respectively. *H*_0_(*t*) is the single-particle Hamiltonian,


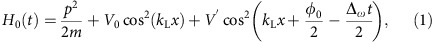


where the second and the third terms describe the static and moving optical lattices, respectively, and *φ*_0_ is the initial relative phase between the two sets of lattices.

When the static lattice depth *V*_0_ is large and when |Δ_*ω*_| is close to the energy gap *E*_*sp*_, higher orbital bands are not significantly populated in the driven process and the system is well described by a simple two-band tight-binding model^38^. Following the standard procedure in Floquet theory, we obtain the effective single-particle Hamiltonian (see Methods)


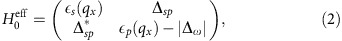


where


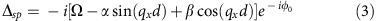


is the coupling between *s*- and *p*-orbital bands that is induced by the moving lattice potential for Δ_*ω*_>0 (see Methods for more details), and 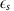 and 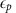 are the energy dispersions for the uncoupled orbital bands. The three coupling coefficients Ω, *α* and *β* are given by 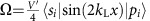, 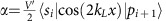 and 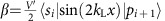, where |*s*_*i*_〉 and |*p*_*i*_〉 are the maximally localized Wannier orbital states in the *i*th site. Ω is the coupling between *s*- and *p*-orbital states in the same lattice site, while *α* and *β* are the couplings between *s*- and *p*-orbital states of nearest neighbouring sites. SMC between *s*–*p* band psuedospins is represented by *α* sin(*q*_*x*_*d*)*σ*_*x*_ after a spin rotation.

This derivation shows that the inversion symmetry of the FB band structure is broken due to the coexistence of couplings of different parities. When the moving lattice intensity is adiabatically ramped on, the quasimomentum of the BEC gradually shifts away from *q*_*x*_=0 in a definite direction following the hybrid band minimum. This is quite different from previous shaken lattice experiments^17^, where the inversion symmetry of the band was preserved and the BEC could spontaneously choose either side of *q*_*x*_=0 as its ground state. In that case, the BEC needed to be accelerated to break the inversion symmetry. In our scheme, the position of the true minimum is uniquely determined by the moving velocity direction, moving lattice depth and driving frequency.

This minimal two-band model captures the essential physics of the driven lattices, as we have seen through the comparison of experimental measurements and theoretical values (Figs 2 and 3), demonstrating the observation of SMC between *s*–*p* band pseudospins. However, this model may deviate from the experiment when the modulated dynamics involve additional orbital bands or when the nonlinear interaction is strong such that the single-particle band structure will be renormalized by the interaction term.

### Quench dynamics

**Figure 5:**
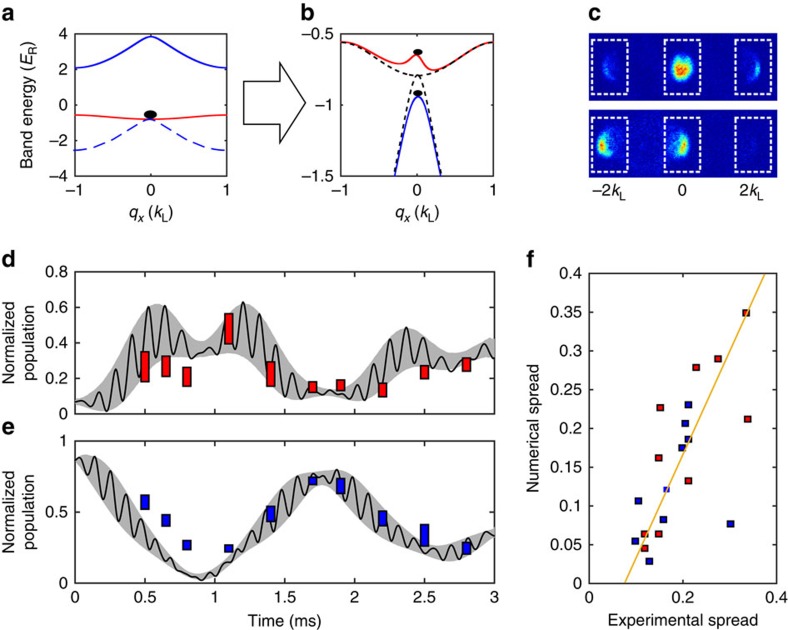
Quench dynamics after suddenly jumping on the coupling between the *s* and *p* band. Band structure before (**a**) and after (**b**) jumping on the coupling between the *s* and *p* band. (**c**) Two examples for experimental images taken 0.5 ms after the quench. The difference between the images is due to the relative phase between the static and the moving lattice. The experimental data for **d** and **e** are extracted from such images by counting the atom number in the dashed boxes. They indicate the −2*ħk* (left rectangle), 0 (middle rectangle) and +2*ħk* component (right rectangle). (**d**,**e**) The normalized population of the momentum component −2*ħk* and 0 *ħk,* respectively. The spread of the experimental data, indicated by the height of the rectangles, is the s.d. of 10 measurements. The shaded areas are the results of numerical GP simulations calculated for a uniform spread of phases *φ*_0_. The black curves show the numerical result for *φ*_0_=0. (**f**) The correlation between numerical and experimental spread for the data from **d** and **e**. The solid line is a least squares fit to the scatter.

Since a Floquet system is generated by a time-periodic Hamiltonian, an important question concerns the role of the initial phase of the driving field^29^. For the system considered in this work, this phase determines the relative positions between the moving and static lattice sites. Though the relative phase does not change the effective band structure (equation (2)), and thus the time-averaged dynamics, it can play a crucial role in the micromotion of the BEC. To demonstrate the effect of the initial relative phase, we study the oscillations in the population of the momentum components *k*_*x*_=0, ±2*k*_L_ after a quantum quench. Figure 5 presents such quench dynamics after adiabatically ramping on the static lattice to 5.47 *E*_R_ followed by a sudden jump on of the moving lattice to 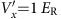 with an on-resonant driving frequency |Δ_*ω*_|=*E*_*sp*_. Figure 5a,b shows the effective band structure before (Fig. 5a) and after (Fig. 5b) the moving lattice is jumped on. The jump projects the BEC on to the new band structure and the BEC acquires components in the new lower and upper hybrid band. Subsequently, these components beat against each other. We focus on the evolution during the first 3 ms, during which the BEC mainly stays at *q*_*x*_=0 without significant dipole motion in the hybrid bands. The TOF images consist of three momentum populations as shown in the two examples in Fig. 5c, which are both taken after 0.5 ms of evolution. In our experiments, the initial phase *φ*_0_ between the static and the moving lattice is uncontrolled, and is different in each repetition of the experiment. This leads to different population dynamics in each experimental iteration and explains the differences between the two images in Fig. 5c. The population dynamics measured in 10 subsequent experimental iterations are represented by the rectangles in Fig. 5d,e. For each measurement time, the height of the rectangle indicates the spread in the data. The shaded areas represent the result of numerical GP simulations for a homogeneous spread of relative phases. We find a strong correlation between the experimental spread and the spread predicted by the numerics (Fig. 5d,e). Numerical results calculated for the particular value *φ*_0_=0 are shown by the solid line in Fig. 5d,e. They reveal that for a fixed initial phase there are oscillations on two different time scales. The fast oscillations (with period *T*≈0.12 ms) corresponds to the micromotion of particles under the high-frequency periodic driving, whereas the slow oscillation (with *T*≈1.75 ms) corresponds to the time-averaged effective Rabi oscillations between the two hybrid FB bands. For longer holding times, the periodicity is slightly broken due to a small dipole motion. Figure 5f shows the correlation between numerical and experimental spread for the data in Fig. 5d,e. The straight line is a fit showing the correlation trend. A linear correlation of *ρ*_*X*,*Y*_=0.64 is achieved, where *ρ*_*X*,*Y*_ is the Pearson product-moment correlation coefficient defined by 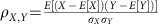. Here *E* is the expectation value and *σ* is the s.d.

## Discussion

We have realized and characterized a SMC with lattice bands as pseudospins. This not only provides a powerful tool to control orbital states with a driving field, but also enriches the study of novel quantum matter using hybrid orbital bands. There are many directions that can be taken along this route, for example, the engineering of similar SMC in higher dimensional systems, involving different orbital bands, and quantitative analysis and measurements of the effects of strong interactions on the effective bands. The realization of similar SMC for fermionic atoms such as ^6^Li and ^40^K with tunable interactions may open the door for exploring exotic quantum matter. For example, for a spin-balanced Fermi gas loaded into such tilted *s–p* dressed bands, the Cooper pairs may acquire a finite centre-of-mass momentum due to the broken inversion symmetry that provides a route to search for the long-sought Fulde–Ferrell–Larkin–Ovchinnikov states^38^. Furthermore, the realized spin–orbit coupling with lattice bands as pseudospins may provide a new platform for the study of topological insulators and Majorana fermions^14^,^15^,^16^.

## Methods

### GP simulations

**Figure 6:**
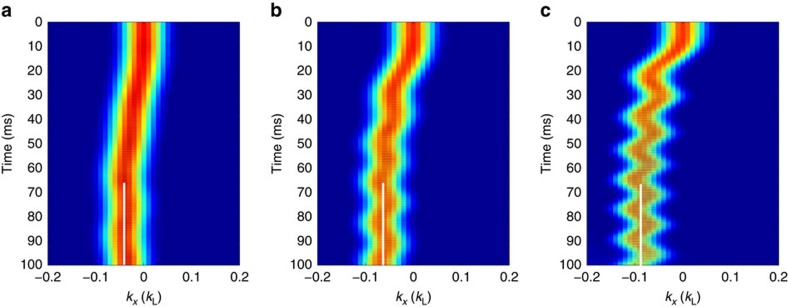
Plots of the evolution of the BEC momentum space density distribution as a function of *k*_*x*_ for different evolution times. The moving lattice depth is ramped to (**a**) *V*′=0.25 *E*_R_, (**b**) *V*′=0.5 *E*_R_ and (**c**) *V*′=1.0 *E*_R_ in the first 60 ms and then keep this value for the following 40 ms. The white lines indicate the centre of the small dipole osicllation from which the band minimum is determined. The driving frequency is |Δ_*ω*_|=5.21 *E*_R_.

In Fig. 6, we present the results of GP simulations for different moving lattice depths. The modification of the band structure minimum can clearly be seen from the shift of the quasimomentum of the BEC during the driving process.

### Tight-binding model

We consider a BEC in a combination of a one-dimensional static optical lattice and a moving lattice,


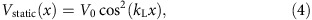



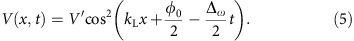


The single-particle Hamiltonian is given by


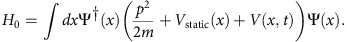


By expanding the wave function in terms of the two lowest orbital states of the static optical lattice and assuming that all other higher orbital bands are not relevant, we obtain


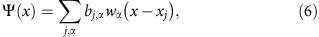


where *α*=*s*, *p* is the orbital index, *j* is the site index and *w*_*α*_(*x*−*x*_*j*_) is the Wannier function for orbital state *α* localized at site *x*_*j*_ with the corresponding annihilation operator *b*_*j*,*α*_. Substituting this wave function into the Hamiltonian, we obtain the tight-binding model


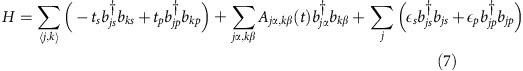



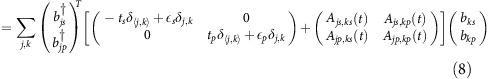


where


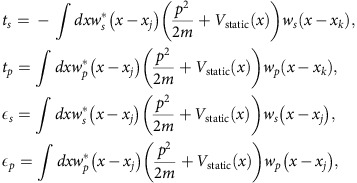


are the bare tunnelling elements between the same orbital states on the adjacent sites |*j*−*k*|=1 (that is, *δ*_〈*j*,*k*〉_=1) and the on-site energies from the static optical lattice. The 〈〉 in the summations indicates summing over the nearest neighbour sites. The moving lattice induces assisted or dressed tunnelling elements between adjacent sites and also modifies the on-site energies:


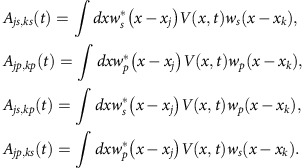


### Time-independent effective Hamiltonian

To obtain a time-independent effective Hamiltonian, we first eliminate the diagonal-dressed terms by a unitary transformation


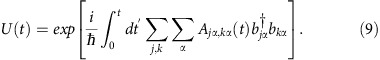


The rotated Hamiltonian is


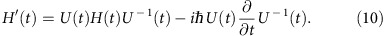


We make the approximation that the driving does not change the on-site energy and bare tunnelling of the same orbital states:


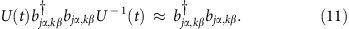


We obtain


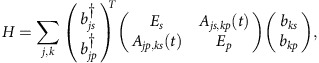


where 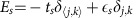 and 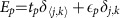. The *s*–*p*_*x*_ couplings induced by the moving lattice are


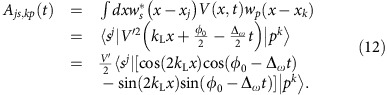


The moving lattice has the same wave vector as the static lattice so that


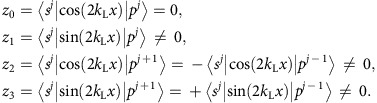


After the Fourier transformation


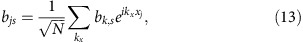



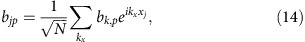


we obtain


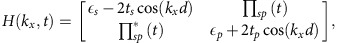


where the *s*–*p* coupling is given by


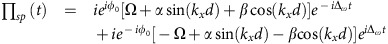


and the coupling coefficients are defined as


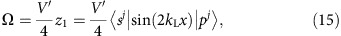



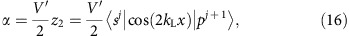



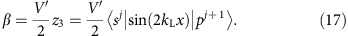


For Δ_*ω*_>0, we perform a unitary transformation followed by the rotating wave approximation,


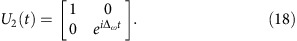


This leads to the time-independent Hamiltonian


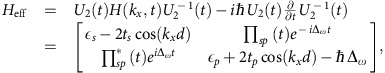


which returns to our effective Hamiltonian equation (2), where we have 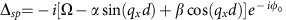. For Δ_*ω*_<0, similar procedures result 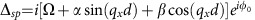. Note that *β* is usually much smaller than Ω and *α*; however, we keep it for completeness.

## Additional information

**How to cite this article:** Khamehchi M. A. *et al.* Spin-momentum coupled Bose-Einstein condensates with lattice band pseudospins. *Nat. Commun.* 7:10867 doi: 10.1038/ncomms10867 (2016).
